# Cross-Reactive Immune Responses toward the Common Cold Human Coronaviruses and Severe Acute Respiratory Syndrome Coronavirus 2 (SARS-CoV-2): Mini-Review and a Murine Study

**DOI:** 10.3390/microorganisms9081643

**Published:** 2021-07-31

**Authors:** Robert E. Sealy, Julia L. Hurwitz

**Affiliations:** 1 Department of Infectious Diseases, St. Jude Children’s Research Hospital, Memphis, TN 38105, USA; bob.sealy@stjude.org; 2 Department of Microbiology, Immunology and Biochemistry, University of Tennessee Health Science Center, Memphis, TN 38163, USA

**Keywords:** SARS-CoV-2, common cold human coronaviruses, cross-reactive antibodies

## Abstract

While severe acute respiratory syndrome coronavirus 2 (SARS-CoV-2) causes serious morbidity and mortality in humans (coronavirus disease 2019, COVID-19), there is an enormous range of disease outcomes following virus exposures. Some individuals are asymptomatic while others succumb to virus infection within days. Presently, the factors responsible for disease severity are not fully understood. One factor that may influence virus control is pre-existing immunity conferred by an individual’s past exposures to common cold human coronaviruses (HCoVs). Here, we describe previous literature and a new, murine study designed to examine cross-reactive immune responses between SARS-CoV-2 and common cold HCoVs (represented by prototypes OC43, HKU1, 229E, and NL63). Experimental results have been mixed. In SARS-CoV-2-unexposed humans, cross-reactive serum antibodies were identified toward nucleocapsid (N) and the spike subunit S2. S2-specific antibodies were in some cases associated with neutralization. SARS-CoV-2-unexposed humans rarely exhibited antibody responses to the SARS-CoV-2 spike subunit S1, and when naïve mice were immunized with adjuvanted S1 from either SARS-CoV-2 or common cold HCoVs, S1-specific antibodies were poorly cross-reactive. When humans were naturally infected with SARS-CoV-2, cross-reactive antibodies that recognized common cold HCoV antigens increased in magnitude. Cross-reactive T cells, like antibodies, were present in humans prior to SARS-CoV-2 exposures and increased following SARS-CoV-2 infections. Some studies suggested that human infections with common cold HCoVs afforded protection against disease caused by subsequent exposures to SARS-CoV-2. Small animal models are now available for the testing of controlled SARS-CoV-2 infections. Additionally, in the United Kingdom, a program of SARS-CoV-2 human challenge experiments has received regulatory approval. Future, controlled experimental challenge studies may better define how pre-existing, cross-reactive immune responses influence SARS-CoV-2 infection outcomes.

## 1. Severe Acute Respiratory Syndrome Coronavirus 2

Severe acute respiratory syndrome coronavirus 2 (SARS-CoV-2) is an RNA virus that was first identified in Wuhan China in December 2019 [1,2]. It has since been the cause of an unprecedented global pandemic. As of 12 July 2021, there were more than 33 million cases of SARS-CoV-2 in the United States and more than 600,000 reported deaths. Globally, there were more than 186 million cases and more than four million deaths [3]. The symptoms of disease (coronavirus disease 2019, COVID-19) usually occur 2–14 days after a virus exposure and can include fever, dry cough, shortness of breath, difficulty breathing, fatigue, body aches, headache, loss of smell/taste, sore throat, nasal congestion, rhinorrhea, loss of appetite, nausea, vomiting and diarrhea. While SARS-CoV-2 is generally recognized as a respiratory disease, viral RNA has been detected in more than 50% stool samples within some patient populations [4]. Individuals over 65 years of age and those with underlying medical conditions including cancer, obesity, chronic kidney disease, heart/lung disease, and diabetes are particularly vulnerable to serious disease caused by SARS-CoV-2. Children are often asymptomatic, but can suffer a rare, serious disease, termed multisystem inflammatory syndrome in children (MIS-C) [5].

Coronaviruses are spherical, enveloped viruses with a positive single strand RNA genome. SARS-CoV-2 is a betacoronavirus, one of four different genera (alpha, beta, gamma, delta) of coronaviruses [6]. The first sequenced SARS-CoV-2 genome, approximately 30 kilobases (kb) in length, is available in the NCBI GenBank database (Accession# NC_045512) [1,2]. Four major structural proteins of the virus are nucleocapsid (N), spike (S), envelope (E), and membrane (M) [6,7]. The N protein forms a complex with viral RNA to form a helical capsid. S, E, and M are all membrane proteins. The S membrane protein supports virus interaction with the target mammalian cell. Two S protein subunits are S1 and S2. The S1 subunit includes the receptor binding domain (RBD) that links virus to its host receptor, the angiotensin-converting enzyme 2 (ACE2) on mammalian cells, and the S2 subunit supports fusion of virus with the mammalian cell membrane [6]. The E protein forms a cation-selective channel and mediates virus budding and release [8]. The M protein contributes to virus assembly and budding. The expression of M with E is sufficient to form virus-like particles in the absence of other viral proteins and RNA [9].

Vaccines have been rapidly developed and released for the prevention of SARS-CoV-2 [10,11,12,13,14], but as of July 2021, vaccines were received by only a fraction of the world population. The pandemic of SARS-CoV-2 continues today as scientists and community leaders strategize to administer vaccines worldwide and quell the spread and evolution of virus. During the pandemic, SARS-CoV-2 variants of concern (VOC) have gained dominance in certain human populations. Some VOCs include B.1.1.7, B.1.351, P.1, and B.1.617.2 (also known respectively as alpha, beta, gamma and delta variants). VOCs were first detected in the United Kingdom, South Africa, Brazil, and India, but quickly spread beyond the countries of origin. Debates are ongoing as to whether current vaccines are adequate to cover VOCs or whether vaccines must be altered to combat new viral mutations [15].

## 2. Common Cold Human Coronaviruses (HCoV)

The common cold HCoVs are usually associated with mild disease in the human population [16,17]. Serological studies suggest that most individuals have been exposed to the common cold HCoVs [18]. Typically, disease is restricted to the upper respiratory tract and virus is rapidly cleared. Symptoms may include rhinitis, pharyngitis, sneezing, hoarseness, and cough [19]. However, common cold HCoVs can also cause serious disease consequences. Advanced age, pre-existing heart and/or lung conditions, and immunodeficiencies can render patients particularly vulnerable to severe disease outcomes [20].

The common cold HCoVs are represented by four viral prototypes, originally categorized serologically. There are contrasting reports in the literature concerning the first isolation of these four strains [6,21,22]. The alphacoronaviruses are NL63 and 229E. The first isolation of NL63 was reported to have occurred in the Netherlands with a sample derived from a child with bronchiolitis [23,24,25]. The first isolation of 229E was reported to have been by Hamre and Procknow who passaged specimens from students with respiratory symptoms at the University of Chicago, USA [22]. Almeida and Tyrrell in the United Kingdom later received 229E from Hamre for further analyses and comparison to other virus isolates (e.g., B814) [26,27,28,29,30,31]. OC43 and HKU1, like SARS-CoV-2, are betacoronaviruses. The first isolation of OC43 was reported to have been by McIntosh et al. at the National Institutes of Health, USA. These investigators used specimens from patients with common cold symptoms and employed an embryonic trachea organ culture method that was previously developed by Tyrell and colleagues to isolate virus [26,32,33,34,35]. HKU1 was reported to have derived from a hospitalized adult man with chronic pulmonary disease in Hong Kong [25,36,37,38]. 229E and OC43 were first described in the 1960s, while NL63 and HKU1 were first described in 2004–2005 [39]. In early years, electron microscopy studies were used to characterize viral morphology, revealing a crown-like structure or ‘corona’ on each of the isolates [21]. SARS-CoV-2 is better related to OC43 and HKU1 than NL63 and 229E by sequence (sequence alignments and phylogenetic trees of coronaviruses are described by Gussow et al.) [40]. SARS-CoV-2 and NL63 are related in that they share ACE2 binding [6,22,25,32,34,36,38,39,41,42,43,44,45].

## 3. Human Responses toward SARS-CoV-2 in SARS-CoV-2 Unexposed Individuals

Given that many individuals have been exposed to common cold HCoV and have generated HCoV-specific B cell and T cell responses [46,47], a pertinent question is whether common cold HCoV-specific antibodies and T cells cross-react with SARS-CoV-2. Research has yielded variable results [17,47,48,49,50,51,52,53,54,55].

In order to address the question, one strategy was to examine sera from SARS-CoV-2-unexposed individuals, usually acquired before the SARS-CoV-2 pandemic began. Researchers asked if these ‘pre-pandemic’ samples contained SARS-CoV-2-specific antibodies. The results of a few reports are shown in Table 1. As shown, there were variable levels of SARS-CoV-2-specific antibodies found in SARS-CoV-2-unexposed individuals. Common antibody targets were N and S2. In some cases, S2-specific antibodies were associated with neutralization.

In a separate report by Shiakolas et al. [51], human monoclonal antibodies with SARS-CoV-2 S binding were synthesized and studied to test for cross-reactive potentials. Antibody variable region sequences were from a blood donor who had been exposed to SARS-CoV-1 more than ten years prior to sample collection (potential exposures to other coronaviruses were not reported). The procedure began by purification of donor B cells based on their binding to at least one coronavirus S antigen within an antigen pool. This was followed by the sequencing of corresponding immunoglobulin genes using LIBRA-Seq (a high-throughput sequencing program designed to link B cell receptors with antigen specificities), and the selection of variable gene sequences for expression in custom plasmids. Six monoclonals were generated that bound SARS-CoV-1 S and SARS-CoV-2 S. Two of these monoclonals also bound HKU1 S and OC43 S, albeit weakly. One of the two antibodies with HKU1 S and OC43 S binding also bound the N terminal domain of SARS-CoV-2 S1 while the second monoclonal bound the SARS-CoV-2 RBD. The RBD-specific monoclonal supported Fc effector functions including antibody-dependent cellular phagocytosis in vitro, but no significant neutralization. In an in vivo experiment with a mouse modified SARS-CoV-2 challenge, the RBD-specific antibody did not significantly reduce viral loads or improve animal survival, but reduced pulmonary hemorrhage (scored by lung color at the time of mouse sacrifice).

Altogether, the studies of SARS-CoV-2-unexposed persons revealed antibody responsiveness toward SARS-CoV-2 with variable frequencies and binding targets. Many studies were with sera, not monoclonal antibodies, in which case it was surmised, but unproven, that individual antibodies bound both common cold HCoVs and SARS-CoV-2, and that the induction of cross-reactive antibodies was due to previous common cold HCoV exposures. As an alternative explanation, one author suggested that pre-pandemic antibodies responsive to SARS-CoV-2 might have been induced by other antigens such as those in vaccines for diptheria, polio, and tetanus (DPT) [64].

## 4. Upregulation of Cross-Reactive Antibodies after SARS-CoV-2 Infections

As a parallel strategy to the analyses of samples from SARS-CoV-2-unexposed individuals, researchers asked if SARS-CoV-2-exposed individuals (often identified by a positive SARS-CoV-2 polymerase chain reaction, PCR), when compared to SARS-CoV-2-unexposed individuals, exhibited enhanced responses to the common cold HCoVs. Samples of reports and results are listed in Table 2. As demonstrated, immune responses toward the common cold HCoVs were often upregulated following SARS-CoV-2 exposures suggesting that responses were cross-reactive. Again, antibody targets were often N and S2.

The combined results from testing pre-pandemic sera, post-pandemic sera, and monoclonals told a consistent story. They showed that certain antibodies could cross-react, at least weakly, between SARS-CoV-2 and common cold HCoV proteins and that S (particularly S2, a potential target of neutralization) and N proteins were common targets of antibody binding.

## 5. Testing Cross-Reactive S1 Antibody Induction in a Controlled Research Setting

Cross-reactive antibodies between the common cold HCoV S1 and SARS-CoV-2 S1 in human sera are difficult to detect [66,67]. The interpretation of these results is complicated, because the full histories of pathogen exposures are unknown. The timing and origins of immune responses may be incorrectly assigned. Co-infections further complicate data interpretations. Small animals, although non-identical to humans, provide a controlled setting for analyses of immune responses. Mice, like humans, have sophisticated immunoglobulin loci comprising variable (V), diversity (D), joining (J), and constant (C) region genes, and generate an extraordinary number of unique antibodies via gene segment recombination. In numerous instances, murine antibodies have been instrumental in the definition of viral protein structures, antibody targets, and amino acid sequences that are pertinent to antibody cross-reactivity [68,69,70]. We thus designed controlled murine experiments to test whether cross-reactive SARS-CoV-2 S1-specific antibodies could be deliberately induced by immunizations with S1 from each of the common cold HCoVs.

Groups of C57BL/6 mice were immunized with S1, either from SARS-CoV-2 or from each of the four common cold HCoVs (OC43, NL63, 229E, and HKU1). A separate set of mice received the RBD of SARS-CoV-2, and control mice received phosphate buffered saline (PBS) with no antigen. Three mice per group were used in each of two independent experiments. The first immunization was with 5 μg protein emulsified in complete Freunds adjuvant (CFA, 1:1, Thermo Scientific), administered in 100 μL intraperitoneally (IP). A booster 21 days later was with 5 μg of the same protein in incomplete Freunds adjuvant (IFA, Thermo Scientific). Mice were bled on day 15 post-boost and sera were stored at −20 °C prior to testing in an ELISA.

**Figure 1:**
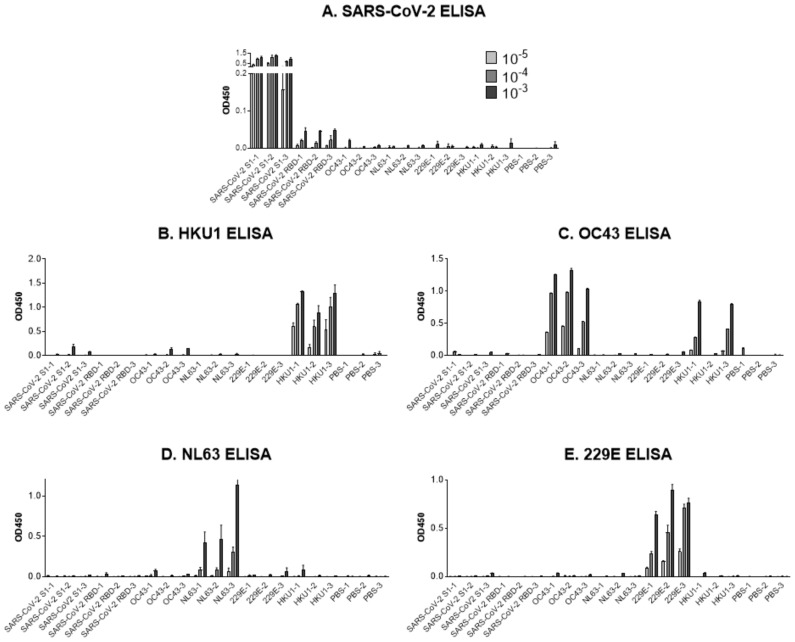
Poor cross-reactivity between recombinant S1 proteins from SARS-CoV-2 and common cold HCoVs. Method: Mice were female C57Bl/6J from Jackson Laboratories. Animal experiments were reviewed and approved by the St. Jude Institutional Animal Care and Use Committee (IACUC). Individual mouse sera (X axes) were named by immunogen and mouse number (1,2, or 3, for each of three mice per immunization group). All antigens were S1 except for the SARS-CoV-2-RBD. PBS-primed mice were negative controls. Recombinant spike proteins used for priming and ELISAs were obtained from SINO Biologicals and were produced from HEK293 cells (unless indicated below). Proteins were tagged at the C terminus. Proteins included common cold HCoV-HKU1 (40021 V08H, S1 amino acids (aa) Met1-Arg760 with a polyhistidine (His) tag), 2019nCoV (SARS-CoV-2, 40591 V08H, S1 a.a. Val16-Arg685, His-tag), common cold HCoV-OC43 (40607 V08B, S1 and S2 aa Met1-Pro1304, expressed in baculovirus insect cells), common cold HCoV-NL63 (40600 V08H, S1 aa Cys19-Val717, His tag) common cold HCoV-229E Spike S1 (40601 V08H, S1 aa Cys16-Asn536, His tag), and 2019nCoV (SARS-CoV-2) RBD (40592 V05H, S1 aa arg319-phe541, tagged with the Fc region of mouse IgG1). To perform the ELISA, each S1 protein (excluding the SARS-CoV-2 RBD, 40592 V05H) was coated separately overnight (0.5 μg/mL in 100 μL PBS) on flat bottomed 96 well plates. Plates were washed three times with Dulbecco’s phosphate buffered saline (DPBS) and blocked with 100 μL 1% bovine serum albumin (BSA) for 1 h at 37 °C. Mouse sera were diluted in 1% BSA, 0.05% TWEEN 20 in PBS and 100 μL were added per well. Plates were incubated for 30 min at 37 °C and washed with 0.05% TWEEN 20 in PBS. Then 100 μL goat anti-mouse IgG H + L antibody conjugated to horse radish peroxidase (HRP, Southern Biotechnologies 1031-05; diluted 1:5000 in 1% BSA in PBS) were added and incubated for 30 min at 37 °C. Plates were washed with 0.05% TWEEN 20 in PBS. Plates then received 100 μL TMB substrate (KPL) and reactions were stopped with 100 μL 1M H_3_PO_4_ within 5 min of reagent addition. Plates were read within 15–20 min after reactions were stopped. OD_450_ nm readings are shown with serum dilutions of 1:1000, 1:10,000 and 1:100,000 (see graph legend). Plates were coated with S1 proteins from (**A**) SARS-CoV-2, (**B**) HKU1, (**C**) OC43, (**D**) NL63, and (**E**) 229E.

ELISAs were performed with mouse sera to test for binding toward the array of S1 proteins (excluding the RBD of SARS-CoV-2). Representative results are shown in Figure 1 with serum from each mouse (listed on the X axis) tested for binding with each S1 protein. ELISA plates were coated with S1 proteins from A. SARS-CoV-2, B. HKU1, C. OC43, D. NL63, and E. 229E. While each S1 vaccine generated antibody responses toward the homologous antigen, the cross-binding potentials were poor in most instances.

Mice vaccinated with the RBD of SARS-CoV-2 generated a response, albeit weak, toward the SARS-CoV-2 S1 recombinant (Figure 1A). Mice primed with S1 proteins of OC43, NL63, 229E, and HKU1 all exhibited a poor response toward SARS-CoV-2 S1 (Figure 1A). The HKU1 S1 protein induced significant antibodies toward OC43 S1 in some (but not all) mice, reflecting similarities between these two betacoronaviruses (Figure 1C). Similar to previous findings, we found that OC43 and 229E S proteins were poorly related serologically [19]. Our finding of poor induction of SARS-CoV-2 S1-specific antibodies by common cold HCoV S1 proteins extended the finding of Kim et al. who demonstrated that SARS-CoV-2 S1-primed mice generated negligible responses toward NL63 [71].

When reviewing in vitro data, we emphasize that assay targets were recombinant, truncated and secreted proteins that did not fully represent the three-dimensional and four-dimensional (3D and 4D) structures of S1 in an infected cell. The assays were therefore unlikely to detect every S1-specific antibody. Nonetheless, the results supported the use of S1 sequences in assays designed to diagnose SARS-CoV-2 infections specifically [72]. The results also supported previous literature showing that cross-reactive antibody responses between S1 proteins of SARS-CoV-2 and the common cold HCoVs were difficult to detect.

## 6. T Cells Cross-React with Common Cold HCoVs and SARS-CoV-2

A number of cross-reactive T cell responses and epitopes have been mapped between SARS-CoV-2 and the common cold HCoVs [73,74,75,76,77,78,79]. Le Bert et al. [75], for example, reported frequent T cell responses to SARS-CoV-2 non-structural and N proteins among SARS-CoV-2-unexposed donors. As was the case for antibodies, reports of virus-specific T cell frequencies have varied between studies based on host group and assay. Both CD8+ and CD4+ T cell cross-reactive populations have been identified, and for each population, cross-reactive peptide epitopes have been described [73,74,75,76,77,78,80,81]. T cells may exhibit a variety of effector functions toward SARS-CoV-2 or common cold HCoVs including the secretion of cytokines/chemokines, the killing of SARS-CoV-2 infected cells (cytotoxic T lymphocyte, CTL), the provision of help by cognate interactions with B cells (T helper [TH] or T follicular helper [TFH]), and/or the targeted down-regulation of an immune response (Regulatory T cell, Treg). In some studies, more than 50% of humans with no known previous exposures to SARS CoV-2 have exhibited T cell reactivity toward SARS-CoV-2 [48]. Cross-reactive T cells, like B cells, were upregulated upon exposures to SARS-CoV-2.

A point to be considered when examining T cells is that ‘help’ from a SARS-CoV-2 specific T cell can be relayed to a SARS-CoV-2-specific B cell when B cell and T cell epitopes do not match. For example, a T cell that recognizes an N peptide of SARS-CoV-2 could suffice to ‘help’ a B cell that produces S-specific, neutralizing antibodies. This is because a B cell with antibodies that bind viral surface proteins (e.g., S) can internalize virions and process a variety of viral peptides (from both internal and external viral proteins) for presentation with major histocompatibility complex (MHC) class II proteins on the B cell surface. A T cell receptor that recognizes any one of these viral peptides (e.g., N) might then deliver cognate T cell help to an S-specific B cell [82].

## 7. Influence of Cross-Reactive Immune Responses on SARS-CoV-2 Disease in Humans

We ask, what is the outcome of human SARS-CoV-2 infections in the context of pre-existing immunity toward a cross-reactive epitope on common cold HCoVs? Do pre-pandemic cross-reactive immune responses confer benefit or harm? The results have, again, been mixed.

Anderson et al. [46] suggested that pre-pandemic SARS-CoV-2 cross-reactive antibodies were not associated with reducing SARS-CoV-2 infections. This conclusion was based on the finding that groups of SARS-CoV-2-infected and uninfected persons had similar SARS-CoV-2-specific IgG levels when their banked pre-pandemic samples were tested. The authors acknowledged that results were not definitive due to experimental weaknesses. Weaknesses included small group sizes, unknown SARS-CoV-2 exposure frequencies (particularly in the uninfected group), low frequencies of S-specific, cross-reactive antibodies in pre-pandemic samples, and large time differences between the collection of pre-pandemic samples and any subsequent, potential SARS-CoV-2 exposures.

In contrast to the results of Anderson et al., Sagar et al. described an improved outcome for individuals who had a recent, documented history of a common cold HCoV infection prior to experiencing COVID-19, compared to controls [83]. Specifically, patients with a recent, documented history of common cold HCoV infections, when hospitalized with COVID-19, had lower rates of intensive care unit admissions and had improved rates of survival. These results suggested that pre-existing immune responses toward common cold HCoV conferred a degree of protection against SARS-CoV-2.

Another indication that the common cold HCoVs may provide a degree of protection against SARS-CoV-2 is that in low-income, high-density regions, where common cold HCoV exposures are presumed to be high, there are relatively high rates of SARS-CoV-2 infections, but low rates of serious disease. For example, in Mumbai, India SARS-CoV-2 infection rates were higher in slums compared to non-slums, but fatality rates due to SARS-CoV-2 were lower in slums compared to non-slums. It has also been observed that poor, highly populated countries have suffered fewer deaths from SARS-CoV-2 per million individuals compared to Western nations [84,85].

Might cross-reactive immune responses cause harm? In the dengue virus field, pre-existing responses toward non-identical viral variants have in some cases worsened disease due to antibody dependent enhancement (ADE) [86]. It is possible that a similar outcome could be experienced in the context of common cold HCoV and SARS-CoV-2 infections [87]. Pre-existing immunity toward a common cold HCoV may be of particular concern in the context of MIS-C, when children suffer disease due to an over-active immune response toward SARS-CoV-2. When a SARS-CoV-2 exposure occurs, as with any virus, the immune response must achieve a fine balance to support virus clearance without immunopathology. When high-quality pre-existing immunity exists, B cells and T cells may clear virus in the URT before virus amplifies and progresses to the LRT. In this case, the virus infection may be asymptomatic and go unnoticed. In contrast, if the immune response is delayed and virus exists at high titers in the LRT, immune effectors can be detrimental. In this instance, a vigorous and rapid immune response may cause a cytokine storm and irreparable damage to the airways even after replication-competent virus has been cleared. A most notable illustration of immunopathology in the context of a respiratory virus infection was in the 1960s: a formalin-treated respiratory syncytial virus (RSV) vaccine was tested in children, but failed to induce neutralizing antibodies. Instead, immune responses caused significant morbidity and two deaths among vaccinated participants when participants were naturally exposed to RSV at a later date. Researchers are currently debating the extent of immunopathology in patients with COVID-19. Some assume that cytokine storms are prominent. Others argue that few patients with COVID-19 exhibit cytokine profiles indicative of a cytokine storm and that COVID-19 patients are much less inflamed than patients with influenza. Arguments regarding disease symptoms fuel additional debates regarding COVID-19 treatments, as clinicians strive to support virus clearance without immunopathology [88].

One difficulty with human studies is the failure to detect all SARS-CoV-2 exposures. If an individual is protected from SARS-CoV-2 due to a previous exposure to a common cold HCoV, a subsequent SARS-CoV-2 exposure may go unnoticed. Experimental human challenges with common cold HCoVs have already been performed [89,90] and new studies of experimental human SARS-CoV-2 challenges have recently received regulatory approval in the United Kingdom [91]. Perhaps the results from controlled challenge studies in small animal models and human adults [92,93,94] will help better define if/how cross-reactive responses, induced by the common cold HCoVs influence SARS-CoV-2 infections and disease.

## 8. Why Has the Study of Cross-Reactive Immune Responses Yielded Conflicting Messages?

Researchers have often reported contrasting results regarding measurement of cross-reactivity between SARS-CoV-2 and the common cold HCoVs. Some authors have detected no or few cross-reactive antibodies, whereas others have identified antibodies and precise epitope targets. Some authors have observed neutralization capacities and others have not. These discrepancies are a consequence of differences in research subjects (inclusive of small research animals, non-human primates, and humans of various ages, sexes and geographical locations), assays and assay cut-off values. Assays have not yet been standardized, and can vary between laboratories with regard to target antigens, developing reagents, protocols, and interpretations. When designing immune assays, the integrity of target epitopes must be considered. Antibodies often respond to three- or four-dimensional structures that cannot be matched by truncated peptide fragments [69]. For T cell responses, the position of a target peptide within its viral protein and antigen presentation events must also be considered. Even when a target peptide is known to exist within a viral protein, the success of antigen processing for T cell recognition will depend on peptide context [95]. Having these caveats in mind, researchers must view published scientific data with an understanding that assessments of cross-reactive antibodies, T cells, and effector potentials remain incomplete.

## 9. Conclusions

Research of cross-reactive antibodies between common cold HCoVs and SARS-CoV-2 has yielded mixed results. In a significant fraction of SARS-CoV-2-unexposed humans, cross-reactive T cells and antibodies that recognized both common cold HCoVs and SARS-CoV-2 were found. Additionally, when humans were naturally exposed to SARS-CoV-2, there were increases in immune responses toward the common cold HCoVs. Cross-reactive antibodies were frequently observed toward S2 (in some cases associated with neutralizing function) and N, but rarely S1. When mice were deliberately immunized with S1 recombinant proteins, the induction of cross-reactive antibody responses was poor. The presence of cross-reactive antibody responses and cross-reactive T cell responses in humans may well impact diagnoses, prophylaxes, treatments, and outcomes of SARS-CoV-2 infections.

## Figures and Tables

**Table 1:** Antibody activities in humans with no known SARS-CoV-2 exposures.

Authors	Manuscript Title	Results	Paper
Khan et al.	Cross-reactivity between common human coronaviruses and SARS-Cov-2 using coronavirus antigen microarray	Khan et al. used pre-pandemic sera and antigen microarrays to study antibody responses. They observed IgG reactivity toward each of the common cold HCoVs in four of five tested serum samples, but only weak IgG reactivity toward SARS-CoV-2 in all five samples. Weak signals were directed toward SARS-CoV-2 N and S2 antigens.	[47]
Guo et al.	Profiling early humoral response to diagnose novel coronavirus disease (COVID-19)	Guo et al. did not observe SARS-CoV-2 N-specific antibodies within pre-pandemic samples that exhibited positive antibodies toward N proteins of NL63, 229E, OC43, and HKU1.	[56]
Mveang Nzoghet al.	Evidence and implications of pre-existing humoral cross-reactive immunity to SARS-CoV-2.	Mveang Nzoghe et al. tested samples from healthy volunteers taken in 2014. Of 135 samples, 32 (23.7%) tested positive for antibodies against SARS-CoV-2 N.	[57]
To et al.	Seroprevalence of SARS-CoV-2 in Hong Kong and in residents evacuated from Hubei province, China: a multicohort study.	To et al. found that in a Hong Kong population presumed to be SARS-CoV-2-unexposed, 53 of 1938 samples (2.73%) were positive for antibody binding in a SARS-CoV-2 enzyme-linked immunosorbent assay (ELISA).	[17,58,59]
Shrock et al.	Viral epitope profiling of COVID-19 patients reveals cross-reactivity and correlates of severity.	Using VirScan technology, Shrock et al. observed antibodies to SARS-CoV-2 ORF-1, but relatively weak or no activity toward SARS-CoV-2 S or N among 190 pre-pandemic samples.	[60]
Anderson et al.	Seasonal human coronavirus antibodies are boosted upon SARS-CoV-2 infection but not associated with protection.	Anderson et al. reported that in pre-pandemic samples taken in 2017, there was antibody binding to SARS-CoV-2 full-length S in 5.4% samples and antibody binding to SARS-CoV-2 RBD of the S protein in 2% of the samples. SARS-CoV-2 N-specific antibodies were detected in 18.6% of the samples. In this case, antibodies were non-neutralizing.	[46]
Tso et al.	High prevalence of pre-existing serological cross-reactivity against severe acute respiratory syndrome coronavirus-2 (SARS-CoV-2) in sub-Saharan Africa.	Tso et al. identified higher frequencies of SARS-CoV-2-specific antibodies in their study of pre-pandemic samples from individuals in African countries compared to individuals in the USA. Specifically, the prevalence of serological activity against SARS-COV-2 in assays was 19% among samples from Tanzania and 14.1% among samples from Zambia, but only 2.4% among samples from the USA. Among the samples from Africa, responses toward N were higher than those toward S.	[61]
Nguyen-Contant et al.	S protein-reactive IgG and memory B cell production after human SARS-Cov-2 infection includes broad reactivity to the S2 subunit.	Nguyen-Contant et al. examined 21 pre-pandemic samples collected from 2011 to 2014 at the University of Rochester (Rochester, New York, USA). Antibody responses toward SARS-CoV-2 were generally weak. Nonetheless, authors detected serum IgG toward a stabilized ectodomain of SARS-CoV-2 S. The responses were also identified toward SARS-CoV-2 N. Responses toward the SARS-CoV-2 RBD were not identified, but 86% of samples bound SARS-CoV-2 S2. The authors contemplated that responses toward S2 might provide neutralizing activity and a degree of protection against SARS-CoV-2 in humans.	[62]
Ng et al.	Preexisting and de novo humoral immunity to SARS-CoV-2 in humans.	Ng et al. frequently observed SARS-CoV-2 S-specific antibodies in SARS-CoV-2 unexposed individuals. The highest frequencies were observed in children. In a population of SARS-CoV-2-uninfected healthy children between the ages of 1 and 16 years, at least 21 of 48 samples scored positively for SARS-CoV-2 S-specific IgG. Anti-S2 antibodies from SARS-CoV-2-uninfected patients exhibited neutralizing activity against SARS-CoV-2.	[49]
Dalakas et al.	Anti-SARS-Cov-2 antibodies within IVIG preparations: cross-reactivities with seasonal coronaviruses, natural autoimmunity and therapeutic implications.	Dalakas et al. found that the majority of tested, pre-pandemic IVIG preparations (immunoglobulins from pooled serum samples) contained antibodies that cross-reacted with SARS-CoV-2.	[63]
Song et al.	Cross-reactive serum and memory B cell responses to spike protein in SARS-CoV-2 and endemic coronavirus infection.	Song et al. observed minimal or no reactivity to SARS-CoV-2 S among pre-pandemic samples.	[55]

**Table 2:** Antibody responses in patients with COVID-19.

Authors	Manuscript Title	Results	Paper
Nguyen-Contant et al.	S protein-reactive IgG and memory B cell production after human SARS-CoV-2 infection includes broad reactivity to the S2 subunit.	Nguyen-Contant et al. observed upregulation of antibodies toward common cold HCoVs in COVID-19 patients compared to SARS-CoV-2-unexposed controls. As an example, IgG titers toward OC43 S were higher in SARS-CoV-2 convalescent patients compared to unexposed donors. Both S2- and N-specific responses were significantly upregulated in the SARS-CoV-2 -exposed blood donors..	[62]
Shrock et al.	Viral epitope profiling of COVID-19 patients reveals cross-reactivity and correlates of severity.	Shrock et al. used VirScan technology to identify peptide-specific responses that increased in SARS-CoV-2-exposed individuals. They observed elevated antibodies toward peptides in S and N. For example, antibodies toward SARS-CoV-2 S peptides that spanned the region of amino acids 811–830 were increased. An 11 amino acid stretch in this region was found to be highly conserved between SARS-CoV-2 and all four of the common cold HCoVs. This peptide overlapped with the S2 fusion peptide. Additionally, antibodies that targeted another region of the SARS-CoV-2 S2 peptide, amino acids 1144–1163, increased in SARS-CoV-2-exposed individuals compared to unexposed donors. A ten amino acid stretch within this sequence was well matched between SARS-CoV-2 and OC43.	[60]
Yonker et al.	Pediatric Severe Acute Respiratory Syndrome Coronavirus 2 (SARS-CoV-2): Clinical Presentation, Infectivity, and Immune Responses.	Yonker et al. identified improved responses to the RBD of common cold HCoVs (229E, NL63, HKU1, and OC43) in children who were suffering MIS-C due to SARS-CoV2. However, these children also experienced increases in antibodies toward respiratory syncytial virus and influenza virus, suggesting that B cells were activated non-specifically.	[65]
Song et al.	Cross-reactive serum and memory B cell responses to spike protein in SARS-CoV-2 and endemic coronavirus infection.	Authors suggested that pre-existing cross-reactive memory B cells were activated during SARS-CoV-2 infection. Authors identified elevated antibodies toward the HKU1 and NL63 S proteins in SARS-CoV-2-exposed individuals compared to pre-pandemic samples. They also characterized a cross-reactive, S2-specific monoclonal antibody with neutralizing activity. Specifically, single-particle negative stain electron microscopy was used to show that monoclonal CC40.8 bound to the HKU1 S trimer near the bottom of the S2 domain.	[55]
Anderson et al.	Seasonal human coronavirus antibodies are boosted upon SARS-CoV-2 infection but not associated with protection.	Anderson et al. tested samples from 27 hospitalized COVID-19 patients longitudinally and observed increases in antibodies that recognized the S protein of OC43 and SARS-CoV-2 over the course of hospitalization.	[46]

## Data Availability

Additional data may be requested from authors.

## Abbreviations

SARS-CoV-2	severe acute respiratory syndrome coronavirus 2;
HCoV	human coronavirus;
MIS-C	multisystem inflammatory syndrome in children;
RBD	receptor binding domain;
ACE2	angiotensin-converting enzyme 2
